# Differing Lectin Binding Profiles among Human Embryonic Stem Cells and Derivatives Aid in the Isolation of Neural Progenitor Cells

**DOI:** 10.1371/journal.pone.0023266

**Published:** 2011-08-05

**Authors:** Mahesh C. Dodla, Amber Young, Alison Venable, Kowser Hasneen, Raj R. Rao, David W. Machacek, Steven L. Stice

**Affiliations:** 1 Regenerative Bioscience Center, University of Georgia, Athens, Georgia, United States of America; 2 Department of Biochemistry and Molecular Biology, University of Georgia, Athens, Georgia, United States of America; 3 Department of Chemical and Life Sciences Engineering, Virginia Commonwealth University, Richmond, Virginia, United States of America; 4 ArunA Biomedical Inc., Athens, Georgia, United States of America; University of Washington, United States of America

## Abstract

Human embryonic stem cells (hESCs) and their differentiated progeny allow for investigation of important changes/events during normal embryonic development. Currently most of the research is focused on proteinacous changes occurring as a result of differentiation of stem cells and little is known about changes in cell surface glycosylation patterns. Identification of cell lineage specific glycans can help in understanding their role in maintenance, proliferation and differentiation. Furthermore, these glycans can serve as markers for isolation of homogenous populations of cells. Using a panel of eight biotinylated lectins, the glycan expression of hESCs, hESCs-derived human neural progenitors (hNP) cells, and hESCs-derived mesenchymal progenitor (hMP) cells was investigated. Our goal was to identify glycans that are unique for hNP cells and use the corresponding lectins for cell isolation. Flow cytometry and immunocytochemistry were used to determine expression and localization of glycans, respectively, in each cell type. These results show that the glycan expression changes upon differentiation of hESCs and is different for neural and mesenchymal lineage. For example, binding of PHA-L lectin is low in hESCs (14±4.4%) but significantly higher in differentiated hNP cells (99±0.4%) and hMP cells (90±3%). Three lectins: VVA, DBA and LTL have low binding in hESCs and hMP cells, but significantly higher binding in hNP cells. Finally, VVA lectin binding was used to isolate hNP cells from a mixed population of hESCs, hNP cells and hMP cells. This is the first report that compares glycan expression across these human stem cell lineages and identifies significant differences. Also, this is the first study that uses VVA lectin for isolation for human neural progenitor cells.

## Introduction

Human embryonic stem cells (hESCs) can be differentiated into neural progenitor (hNP) cells [1] and mesenchymal progenitor (hMP) cells [2] *in vitro*. hESC-derived hNP cells and hMP cells provide an invaluable source of cells to study early human neural and mesodermal development, respectively, as well as cell replacement therapy, drug screening, and toxicological studies. Human NP cells are likely to follow a similar differentiation pathway as *in vivo* neurogenesis, based on murine studies where early expression of neural markers upon differentiation *in vitro* can parallel that of mouse neurogenesis [3]–[4]. Similarly, hMP cells are likely to follow similar differentiation pathway as adult bone marrow derived mesenchymal stem cells [2], [5]–[6]. The differentiation process allows for investigation of the developmental stages and the associated changes that occur on the cell surface as either a result of or possibly a cause in early differentiation.

Little progress has been made towards defining the carbohydrate surface of hESCs and their differentiated progeny despite recent findings of the importance in cell surface glycoproteins for maintenance of pluripotency, self-renewal, and differentiation. For example, Notch has been implicated in not only directing neural stem cells to a glial cell fate but also neural stem cell renewal. While these two findings may seem contradictory, glysosylation of Notch on different serine residues could cause different outcomes [7]–[8]. Thus, the complexity of the carbohydrate surface has an important role in cell differentiation and renewal. There are several other examples: the role of heparan sulphate proteoglycans in hedgehog signaling [9], chondroitin sulphate proteoglycans in neural migration and differentiation [10]–[13] and recent interest in Galectin-1, a carbohydrate binding protein expressed on mouse neural precursor cells that may affect proliferation of neural stem cells [14]–[15]. The role of the carbohydrate surface is now being brought to the forefront.

Previous studies have characterized the carbohydrate surface of hESCs [16]–[17] and their differentiated progeny in embryoid body differentiation [18]–[19]. However, embryoid body differentiation gives rise to cells of multiple lineages which makes it difficult to characterize each lineage separately. In our laboratory, we have derived highly enriched adherent cultures of human neural progenitor (hNP) cells [1] and human mesenchymal progenitor (hMP) cells [2] by directed differentiation of WA09 hESCs. To ensure a homogeneous population, hESCs, hNP cells and hMP cells were first characterized with pluripotent, neural and mesenchymal intracellular and extracellular markers, previously [1]–[2], and again during this study. The homogenous populations make them ideal for characterization of the cells' carbohydrate expression. A panel of eight lectins was selected based on previous studies [16]–[17] to probe the carbohydrate expression on the cell surface of hESCs, hNP cells and hMP cells. Lectins are carbohydrate binding proteins that recognize diverse sugar structures. We characterized percentages and localization of cells- lectin binding by flow cytometry and immunocytochemistry, respectively. The binding specificity of each lectin was validated using appropriate competitive sugars.

Here, for the first time glycan expression across multiple identified cell types has been compared demonstrating several significant and novel patterns. For example, in hNP cells- lectin binding is enhanced, compared to that of the hESCs and hMP cells, suggesting that increased glycosylation occurs upon neural differentiation *in vitro*; whereas in hMP cells, lectin binding was similar or reduced as compared to that of hESCs. Upon hESCs differentiation to hNP cells and hMP cells, PHA-L lectin binding increases, indicating an up-regulation in expression of complex N-type glycans containing β1–6 linked branches. We also identify that expression of N-acetyl-D-galactosamine (VVA lectin), N-acetyl-D-glucosamine (DBA lectin) and Fucα1,3-GlcNAc glycans (LTL lectin) is significantly up regulated in hNP cells, compared to hESCs and hMP cells. Using the VVA lectin, which binds to N-acetyl-D-galactosamine, we isolated hNP cells from a mixed population of hESCs, hNP cells and hMP cells. This study identifies several extracellular carbohydrate antigens that could be exploited to further characterize and define the hNP cell phenotype.

## Materials and Methods

### Cell culture and passaging

NIH registered hESC line WA09 was used for derivation of differentiated neural progenitor (hNP) cells and mesenchymal progenitor (hMP) cells, as described previously [1]–[2]. Here, to study lectin binding, hESCs were cultured on BD matrigel™ (BD Biosciences) coated 100-mm tissue culture dishes (Techno Plastic Products, Switzerland) and mTeSR®1 medium (Stem Cell Technologies Inc., Vancouver, Canada), according to the company's instructions. The mTeSR®1 culture medium was changed every day and the cells passaged every 4–5 days when the colonies became large and began to merge with each other. For passaging, the culture medium was removed, cells were rinsed with PBS^−−^ (without calcium and magnesium, Gibco Invitrogen) once, and 2 ml of dispase enzyme (1 mg/ml, Stem Cell Technologies Inc.) was added for 7 minutes. Dispase solution was then removed and the cells were rinsed with 4 ml of mTeSR®1 medium once. Fresh mTeSR®1 medium was added again and the colonies were scraped off using a cell scraper (Techno Plastic Products, Switzerland). The cells were passaged 1∶3 or 1∶4 depending on density. The mTeSR®1 medium was changed after two days and then every other day, until ready for passaging again.

The derivation protocols for adherent cultures of hNP cells and hMP cells from hESCs are not discussed here and can be reviewed in previous works [1]–[2]. hNP cells were cultured on BD Matrigel™ coated 100-mm tissue culture treated dishes. Cells were cultured in proliferation medium composed of AB2™ medium (ArunA Biomedical Inc., Athens, GA) supplemented with ANS™ (ArunA Biomedical Inc.), 20 ng/ml Leukimia Inhibitory Factor (LIF, Millipore), 2 mM L- Glutamine, 0.5 U/ml penicillin, 0.5 U/ml streptomycin (all from Gibco Invitrogen), 20 ng/ml FGF-2 (Sigma). Culture medium was changed every other day and hNP cells were passaged every 3–4 days using either a cell scraper or manual pipetting. hNP cells between passage numbers 20–30 were used for this study.

Human mesenchymal progenitor (hMP) cells were cultured in mesenchymal medium consisting of alpha-DMEM, 10% defined fetal bovine serum (FBS, Hyclone), 1 mM L- Glutamine, 0.5 U/ml penicillin, and 0.5 U/ml streptomycin (all from Gibco Invitrogen unless otherwise stated). The culture medium was changed every other day and cells passaged every 3–4 days. For passaging cells from a T-175 flask, 5 ml of 0.05% trypsin, at 37°C, was added and cells incubated at 37°C for 4–5 minutes. To stop the reaction, 2 ml of mesenchymal medium was added. The cells were further dissociated by pipetting 8–10 times with a 10 ml serological pipette. The cell suspension was centrifuged at 1000 RPM for 4 minutes to pellet the cells. The cells were re-suspended in fresh medium and passaged at 1∶2 or 1∶3 dilutions. For this study, hMP cells between passages 7–12 were used.

### Immunocytochemistry of cells for stem cell markers

Immunocytochemistry (ICC) was used to ascertain expression of appropriate markers for the three cell types: hESC (POU5F1^+^/Oct4^+^, SSEA-4^+^, SOX-2^+^, Nestin^−^ and CD166^−^), hNP cells (Oct4^−^, SSEA-4^−^, SOX-2^+^, Nestin^+^ and CD166^−^) and hMP cells (Oct4^−^, SSEA-4^−^, SOX-2^−^, Nestin^+^ and CD166^+^). Four-well chamber slides (BD Biosciences) were used for plating and immunostaining of all the three cell types. WA09 hESCs were passaged onto BD Matrigel™ coated 4-well chamber slides; two days later, the cells were fixed and stained. The hNP cells were collected from 100-mm dish by manual pipetting and plated onto BD Matrigel™ coated 4-well dishes at 50,000 cells/well in fresh proliferation medium. The cells were fixed and stained the next day. hMP cells were passaged from T-175 flasks (BD Falcom) using 0.05% trypsin and plated onto 4-well chamber slides (without Matrigel™) at 50,000 cells/well. The cells were fixed and stained the next day.

All the three cell types were fixed in 2% paraformaldehyde (Fisher Scientific) in 1× PBS^++^ (with calcium and magnesium, Gibco Invitrogen) for 20 minutes. The cells were then washed three times with PBS^++^ and blocked with 1% BSA in PBS^++^ solution for 30 minutes. Primary and secondary antibodies were diluted in the block solution. Cells were then stained with primary antibodies separately for each of the marker proteins: mouse anti-Oct4 (1∶500, Hybridoma), mouse anti-SSEA-4 (1∶20 dilution, Hybridoma), mouse anti-SOX-2 (1∶100, R&D Systems), mouse anti-Nestin (1∶200, Neuromics), mouse anti-CD166 (1∶100, BD Pharmingen) for 1 hr at room temperature. The cells were washed 3 times in PBS^++^ and incubated with secondary antibodies for 1 hr at room temperature. Secondary antibodies included streptavidin conjugated Alexafluor 594 (Molecular Probes; Eugene, OR; 1∶1000 dilution) and goat anti-Mouse IgG conjugated Alexa 488 (Molecular Probes; 1∶1000 dilution). Cells were again washed three times, 5 minutes each, in PBS^++^; and post-stained with 4′-6-Diamidino-2-phenylindole (DAPI, Invitrogen) to detect cell nuclei. Cells incubated with secondary antibodies alone were used as controls. All slides were mounted and visualized using a Nikon TS100 inverted microscope. Individual color channels were captured separately and merged in Adobe Photoshop software (Adobe Inc.).

### Flow cytometry analysis for stem cell markers

The percentage of cells expressing lineage specific markers was determined by flow cytometry. hESCs, hNP cells and hMP cells were harvested as described above, collected in 15 ml sterile conical tubes (Sarstedt Inc.), and fixed in 2% paraformaldehyde in 1× PBS^−−^. The cells were blocked in PBS with 3% fetal bovine serum and 1% BSA for 30 minutes, and placed in sterile conical tubes in aliquots of 500,000 cells for each replicate per antigen. Each cell type was stained with one of the 7 antibodies for 1 hour at room temperature: Rabbit anti-Oct4 (1∶200, Sigma-Aldrich), Goat anti-Sox2 (1∶100, Millipore), Mouse anti-nestin (1∶100, Neuromics), CD73-APC (1∶20), CD166-PE (1∶5), CD90-APC (1∶100), and CD105-FITC (1∶20), all from BD Pharmingen. Cells were washed 3 times with PBS^−−^ and then stained with secondary antibody or isotype control. The secondary antibodies were purchased from Molecular Probes and used at 1∶1000 dilution: donkey anti-mouse APC, mouse anti-goat 488, donkey anti-rabbit PE, and goat anti-rabbit APC. The isotype controls were purchased from BD pharmingen and used 1∶1000 dilution: mouse IgG APC, rat IgM PE, and mouse IgG FITC. Cells stained with secondary antibody only or isotype controls were used as controls. Flow cytometry was performed using CyAn cytometer (Beckman Coulter, Brea, CA) using two lasers – tuned to 488 nm and 633 nm. Data analysis was performed using FlowJo (Tree Star, Inc., Ashland, Oregon). Percentage of cells expressing fluorescence intensity greater than the control cells were calculated using FlowJo program.

### Immunocytochemistry to characterize cell- lectin binding

Table 1 shows the lectins used in this study, their commonly abbreviated name, the specificity of these lectins for their respective oligosaccharide structures and the concentration of hapten, or competitive sugar, that was used to verify specificity of each lectin for its carbohydrate. Specificity of each lectin has previously been described in detail by Cummings and colleagues [20].

**Table 1:** Features of reviewed plant lectins.

Lectin Name	Abbreviation	Oligosaccharide Specificity	Inhibitor
*Concanavalin A*	Con A	N-linked High Mannose or Hybrid, N-linked Di-antennary	200 mM α-methylmannoside orα-methyl glucoside
*Phaseolus vulgaris*leucoagglutinin	PHA-L	N-linked Tri andTetra-antennary withGalβ(1,4)GlNAcβ(1,6)Man	200 mM galactose
*Maackia amurensis* agglutinin	MAA	galactosyl (β-1,4)N-acetylglucosamine	200 mM lactose
*Vicia villosa* agglutinin	VVA	α- or β-linked terminalN-acetylgalactosamine	200 mM N-acetylgalactosamine
*Dolichos biflorus*agglutinin	DBA	(β-1,4) linkedN-acetylglucosamine oligomers	200 mM N-acetylgalactosamine
*Phaseolus vulgaris*erythroagglutinin	PHA-E	N-linked bisected structures	200 mM galactose
*Lotus tetragonolobus*lectin	LTL	Fucα1,3-GlcNAc	50–100 mM L-fucose
*Arachis hypogea*Agglutinin/peanut	PNA	galactosyl (β-1,3)N-acetylgalactosamine	200 mM galactose

The localization of cell surface carbohydrate moieties on routinely maintained adherent cultures of WA09 hESCs, hNP cells and hMP cells was analyzed by immunocytochemistry. hESCs, hNP cells and hMP cells were plated in 4-well chamber slides as mentioned above.

All three cell types were fixed, washed and blocked as mentioned above. Cells were then stained with one of eight biotinylated lectins (Table 1, all lectins obtained from Vector Laboratories; Burlingame, CA; 10 µg/ml in block solution) for 20 minutes at 37°C. The cells were also co-stained with a lineage specific marker: hESCs with mouse anti-SSEA-4 (1∶20 dilution, Hybridoma), hNP cells with mouse anti-Nestin (1∶200, Neuromics); hMP cells with mouse anti-CD166 (1∶100, BD Pharmingen). The cells were washed three times in PBS^++^ and incubated with secondary antibodies for 30 minutes at 37°C. Secondary antibodies included streptavidin conjugated Alexafluor 594 (Molecular Probes; Eugene, OR; 1∶1000 dilution) and goat anti-Mouse IgG conjugated Alexa 488 (Molecular Probes; 1∶1000 dilution). Cells were again washed three times, 5 minutes each, in PBS^++^; and post-stained with DAPI (Invitrogen) to detect cell nuclei. Cells incubated with secondary antibodies alone and without lectins or primary antibodies were used as controls. All slides were mounted and visualized using a Nikon TS100 inverted microscope.

### Flow cytometry analysis to determine percentage cell-lectin binding

The percentage of cells expressing a particular cell surface glycoprotein and binding to corresponding lectin was determined by flow cytometry. hESCs, hNP cells and hMP cells were harvested as described above, collected in 15 ml sterile conical tubes (Sarstedt Inc.), and fixed in 2% paraformaldehyde in 1× PBS^−−^. The cells were blocked in 1% BSA for 30 minutes, and placed in sterile conical tubes in aliquots of 500,000 cells for each replicate per lectin. Each cell type was stained with one of the 8 lectins (Table 1) at 5 µg/ml. Cells were washed 3 times with PBS^−−^ and then stained with secondary antibody, streptavidin -allophycocyanin (1∶250 dilution, BD Biosciences). Cells stained with secondary antibody only were used as controls. Flow cytometry was performed using CyAn cytometer (Beckman Coulter, Brea, CA) using two lasers – tuned to 488 nm and 633 nm. Data analysis was performed using FlowJo (Tree Star, Inc., Ashland, Oregon). Percentage of cells expressing fluorescence intensity greater than the control cells were calculated using FlowJo program. Significance was determined by a Two-way ANOVA and Tukey's Pair-Wise (SAS, Cary, NC, USA) comparisons for each lectin binding for the hESCs, hNP cells and hMP cells. Results where a P-value<0.05 were considered to be significantly different.

### Fluorescence Assisted Cell Sorting based on VVA-fluorescein lectin

Cell sorting was performed using MoFlo cell sorter (Beckman Coulter, Brea, CA) with the laser tuned at 488 nm. Data analysis was performed using FlowJo program (Tree Star,Inc., Ashland, Oregon). The experiment was repeated three times. hESCs, hNP cells and hMP cells were harvested as described above for flow cytometry. The 3 cell types were mixed in 1∶1∶1 ratio and then blocked in 1% BSA for 30 minutes at room temperature. The cell mix was then stained with fluorescein-conjugated VVA lectin at 5 µg/ml for 20 minutes at 37°C. Cells were then washed twice with PBS^−−^ and taken for sorting. Similarly, each of the 3 cell types separately were also stained with fluorescein-conjugated VVA lectin and used as controls.

From the 3 cell mixture, cells having highest expression of fluorescein were sorted out into conical tubes and then plated onto 4-well chamber slides. Next day, the sorted cells (approximately 16 hours later) were fixed and stained, as mentioned above, for expression of Nestin, SOX-2, Oct4, SSEA-4 and CD 166 markers.

## Results

### Characterization of hESCs, hNP cells and hMP cells for stem cell markers

**Figure 1:**
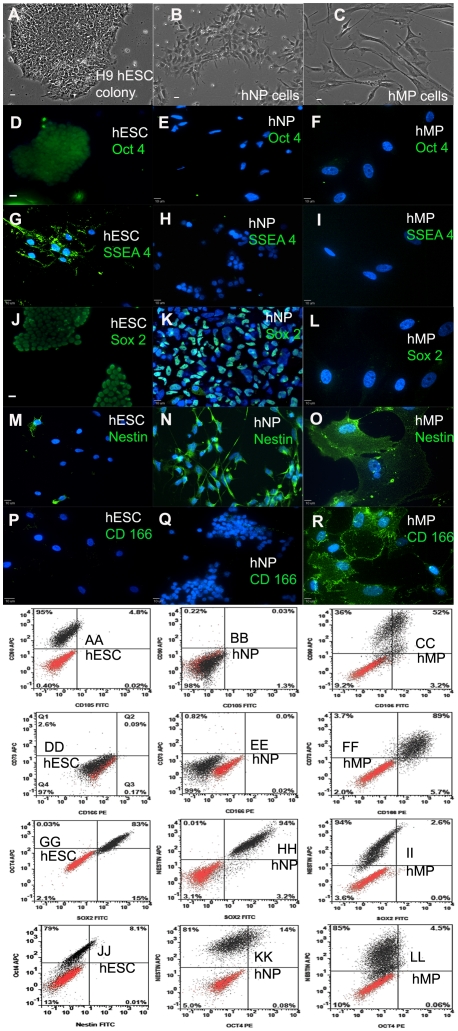
Defining the stem cell phenotype using immunocytochemistry and flow cytometry. Phase contrast image of hESCs (A), hNPs (B), and hMPs (C). hESCs express pluripotency markers: Oct 4 (D,GG, JJ), SSEA-4 (G), and Sox 2 (J,GG); lack expression of Nestin (M, JJ), CD 166 (P,DD), CD73 (DD), and CD105 (AA). hNPs have low expression of pluripotency markers: Oct 4 (E,KK), SSEA-4 (H); and mesenchymal markers CD 166 (Q,EE), CD73 (EE), and CD105 (BB). hNPs express neural markers: Sox 2 (J,HH) and Nestin (N,HH,KK). hMPs lack expression of pluripotency markers: Oct 4 (F,LL), SSEA-4 (I), and Sox 2 (L,II); however, hMPs express Nestin (O,II,LL), CD 166 (R,FF), CD73 (FF), CD90 (CC) and CD105 (CC). All the cells have been stained with the nuclear marker DAPI (blue) in panels D- P. Scale bar: 10 µm. In the dot plots, red dots indicate isotype control or secondary antibody only; black dots indicate the antigen staining.

Cultures of hESCs were maintained on Matrigel™ coated 100-mm tissue culture dishes. hESCs were stained to ensure positive expression for pluripotency markers and negative expression for the lineage specific markers. Most of the hESCs were positive for Oct4 (Figure 1D), SSEA-4 (Figure 1G) and SOX-2 (Figure 1J). The hESCs were negative for Nestin (Figure 1M), a neural marker, and CD 166 (Figure 1P), a mesenchymal marker.

Human NP cells were derived from WA09 hESCs as described previously [1]. hNP cells can be propagated on Matrigel™ coated 100-mm tissue culture dishes for many passages (Figure 1B) and maintain normal karyotype (tested up to 40 passages, data not shown here). These cultures are positive for expression of Nestin (Figure 1N) and SOX-2 (Figure 1K), two main neural stem cell markers, and negative for expression of hESC pluripotency markers Oct4 (Figure 1E) and SSEA-4(Figure 1H); as well as negative for CD 166 (Figure 1Q), a mesenchymal marker.

hMP cells were derived from hESCs as described previously [2] and cultured on tissue culture treated T-175 flasks (Figure 1C). hMP cells were negative for expression of Oct4 (Figure 1F), SSEA-4 (Figure 1I) and SOX-2 (Figure 1L); and positive for the markers Nestin (Figure 1O) and CD 166 (Figure 1R). Cultures of hESCs (SSEA-4^+^), hNP cells (Nestin^+^, SOX-2^+^) and hMP cells (CD166^+^) were stained regularly to ensure that the cultures were homogenous (visual observation) before further analysis.

### Flow Cytometry analysis for expression of stem cell markers

WA09 hESC, hNP and hMP cells were immuno-stained for pluripotent markers (co-staining for Oct4 and Sox2), co-stained for nestin and Sox2; and for the mesenchymal lineage markers CD166, CD105, CD73 and CD90. The cells were then analyzed by flow cytometry. WA09 hESC have high expression of pluripotent markers Oct4 (83%, Figure 1GG) and Sox2 (98%, Figure 1GG), and low expression for nestin (8.1%, Figure 1JJ), mesenchymal markers CD105 (4.8%, Figure 1AA), CD73 (2.6%, Figure 1DD), CD166 (0.26%, Figure 1DD). Human NP cells have low Oct4 expression (14%, Figure 1KK), and high expression of nestin (94%, Figure 1HH, 1KK) and Sox2 (97.2%, Figure 1HH). Human NP cells have low expression for mesenchymal markers CD105 (1.44%, Figure 1BB), CD73 (0.82%, Figure 1EE) and CD166 (0.02%, Figure 1EE). On the other hand, hMP cells have low expression for Oct4 (4.5%, Figure 1LL) and Sox2 (2.6%, Figure 1II); and high expression for mesenchymal markers CD105 (56.2%, Figure 1CC), CD73 (92.7%, Figure 1FF) and CD166 (94.7%, Figure 1FF). Human NP and MP cells both express nestin (Figure 1HH and 1II, respectively).

The flow cytometry analysis shows that the three cell lines, H9 hESC, hNP and hMP, correctly express their lineage markers and have minimal or no cross contamination.

### Analysis of carbohydrate expression by flow cytometry

**Figure 2:**
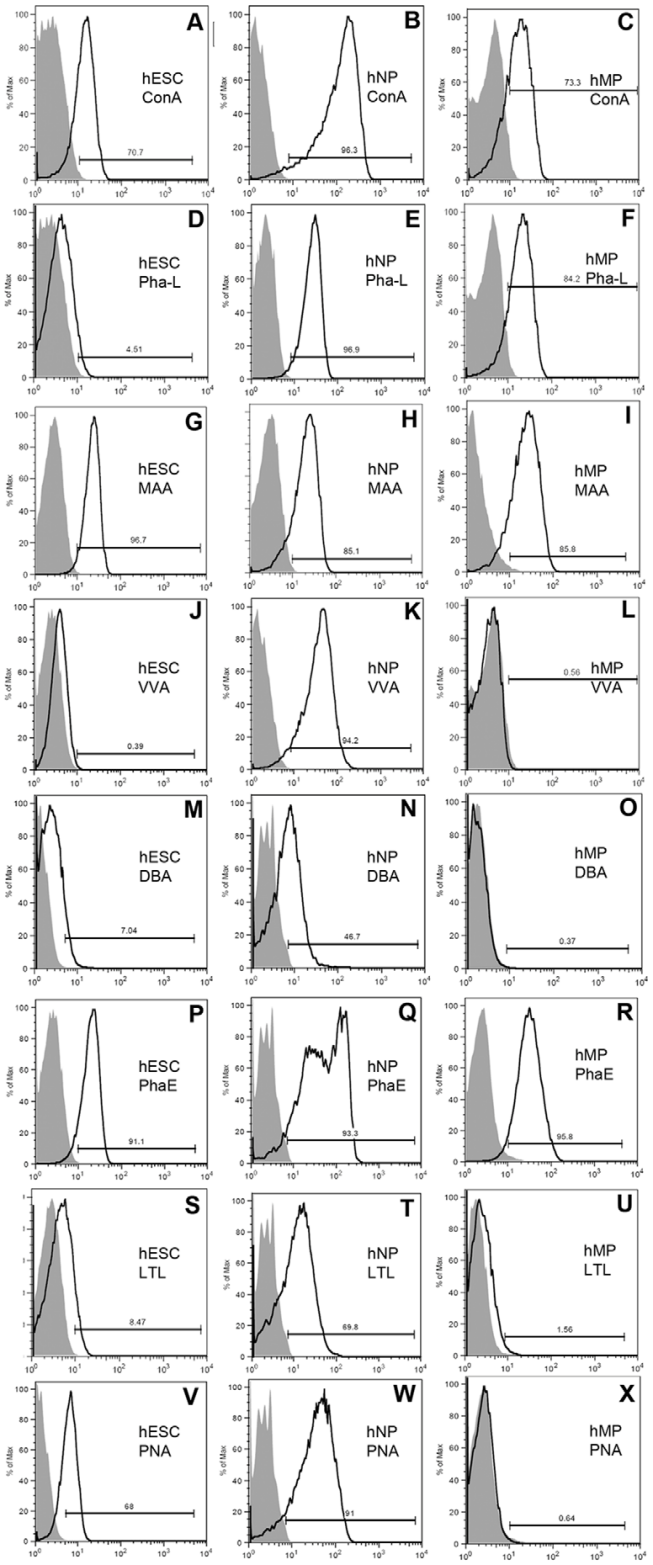
Flow cytometry histograms of lectin binding in hESCs, hNPs and hMPs. Percentage of cells binding to 8 different lectins was determined by flow cytometry. A representative lectin histogram plot is shown for one of 4 experimental replicates. In each panel far left grey fill peak in the histogram plot correlates with cells stained with secondary antibody only, and the shifted black tracing peak represents cells binding to a lectin. Panels in the left column show histograms of 8 different lectins binding to hESCs. Panels in the middle column are for hNP cells and panels in the right column are for hMP cells. Gating for each histogram indicates % of cells positive for the lectin-binding.

**Figure 3:**
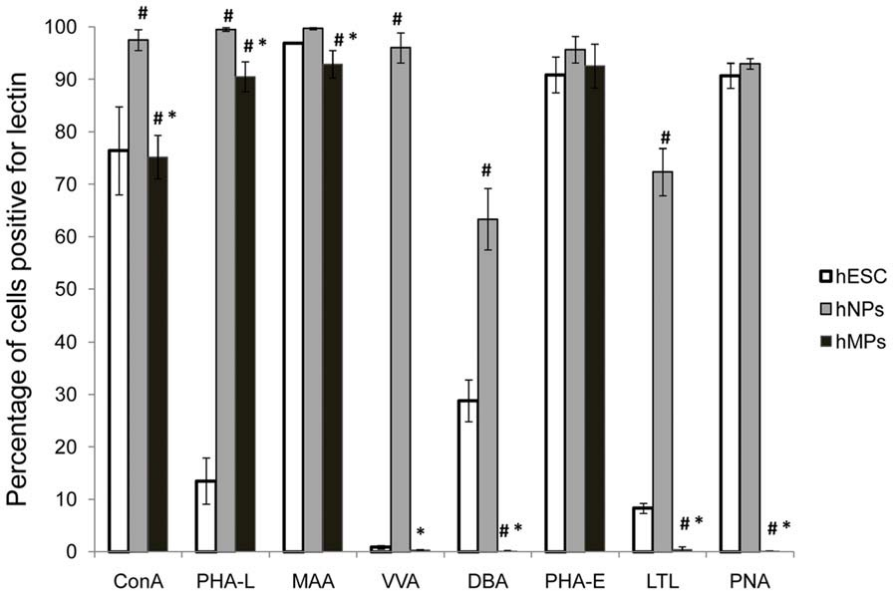
Quantification of lectin binding in hESC, hNP and hMP cell surfaces with 8 different lectins. The percent of cells with specific carbohydrate expression was determined by flow cytometry using 8 different lectins. Each of the 3 cell types were separately stained with one of 8 lectins. The data is represented as average +/− SD of 4 independent assays of hESCs, hNP cells and hMP cells. Means with different letters are significantly different, # indicates p<0.05 compared to hESCs and * indicates p<0.05 compared to hNP cells.

Table 1 shows a list of the lectins used in this study and the oligosaccharides to which they bind. Binding of these lectins represents presence of various carbohydrate structures on the cell surface. Using flow cytometry, analysis of individual histograms of each lectin binding shows peak shifts of lectin-stained cells (black outline histogram, Figure 2) relative to secondary antibody-only (grey-filled histogram, Figure 2) stained cells. Four replicates were completed for each lectin and cell type, experiments were repeated twice and the average values from the 2 repetitions was calculated and analyzed. The lectin-cell binding percentages indicate the percentages of cells positive for lectin binding. A range of lectin binding percentages was observed for the 3 cell types (hESCs, hNP cells and hMP cells) indicating the presence of various glycans on cell surfaces (Figure 3).

First, we consider the hESCs- lectin binding here. Four of the lectins bound to a large percentage of WA09 hESCs, these were MAA (97±0.2%), PHA-E (91±3.4%), PNA (91±2.4%), and ConA (76±8.4%) (Figure 3). Thus, high percentages of hESCs express α-mannopyranosyl (ConA), Neu5Ac residues (MAA), complex N-glycan structures containing GlcNac (PHA-E), and β-galactosyl (PNA). The remaining four lectins bound to fewer WA09 hESCs (p<0.05), these were DBA (29±4%), PHA-L (14±4.4%), LTL (8±1%), and VVA (1±0.4%) lectins. Thus, fewer WA09 hESCs express the glycan structures: complex N-type glycans containing β1–6 linked branches (PHA-L), N-acetyl-D-galactosamine (VVA), α GalNac (DBA) and α(1,2)L-fucose (LTL).

Next, we consider hNP cells- lectins binding here. Five of the eight lectins bound to higher percentage of hNP cells than hESCs (p<0.05), while the remaining three lectin-binding was similar (p>0.05, Figure 3). The lectins and their binding percentages to hNP cells are: PHA-L (99±0.4%), MAA (99±0.2%), ConA (97±2%), VVA (96±3%), PHA-E (96±3%), PNA (93±1%), LTL (72±4.5%), and DBA (63±5.9%). Thus, very high percentages of hNP cells express the glycan structures: α-mannopyranosyl (ConA), complex N-type glycans containing β1–6 linked branches (PHA-L), Neu5Ac residues (MAA), N-acetyl-D-galactosamine (VVA), α GalNac (DBA), complex N-glycan structures containing GlcNac (PHA-E), α(1,2)L-fucose (LTL) and β-galactosyl (PNA).

Finally, we consider hMP cells- lectins binding here. Lectin binding profiles for hMP cells was different compared to hNP cells and hESCs, indicating that cell surface glycosylation pattern is lineage specific. Comparing hMP cells to hESCs (Figure 3), we found that lectin-cell binding percentages were similar for 4 lectins (p>0.05): ConA, MAA, VVA and PHA-E; and thus similar percentages of hESCs and hMP cells express the glycans: α-mannopyranosyl (ConA), Neu5Ac residues (MAA), N-acetyl-D-galactosamine (VVA) and complex N-glycan structures containing GlcNac (PHA-E). Percentage of hMP cells positive for PHA-L lectin binding (thus expression of complex N-type glycans containing β1–6 linked branches) was significantly higher (91±3%, p<0.05) than hESCs. For the rest of the 3 lectins: DBA, LTL and PNA; percentages of cells positive was significantly lower (p<0.05) for hMP cells compared to hESCs.

### Carbohydrate analysis using immunocytochemistry

**Figure 4:**
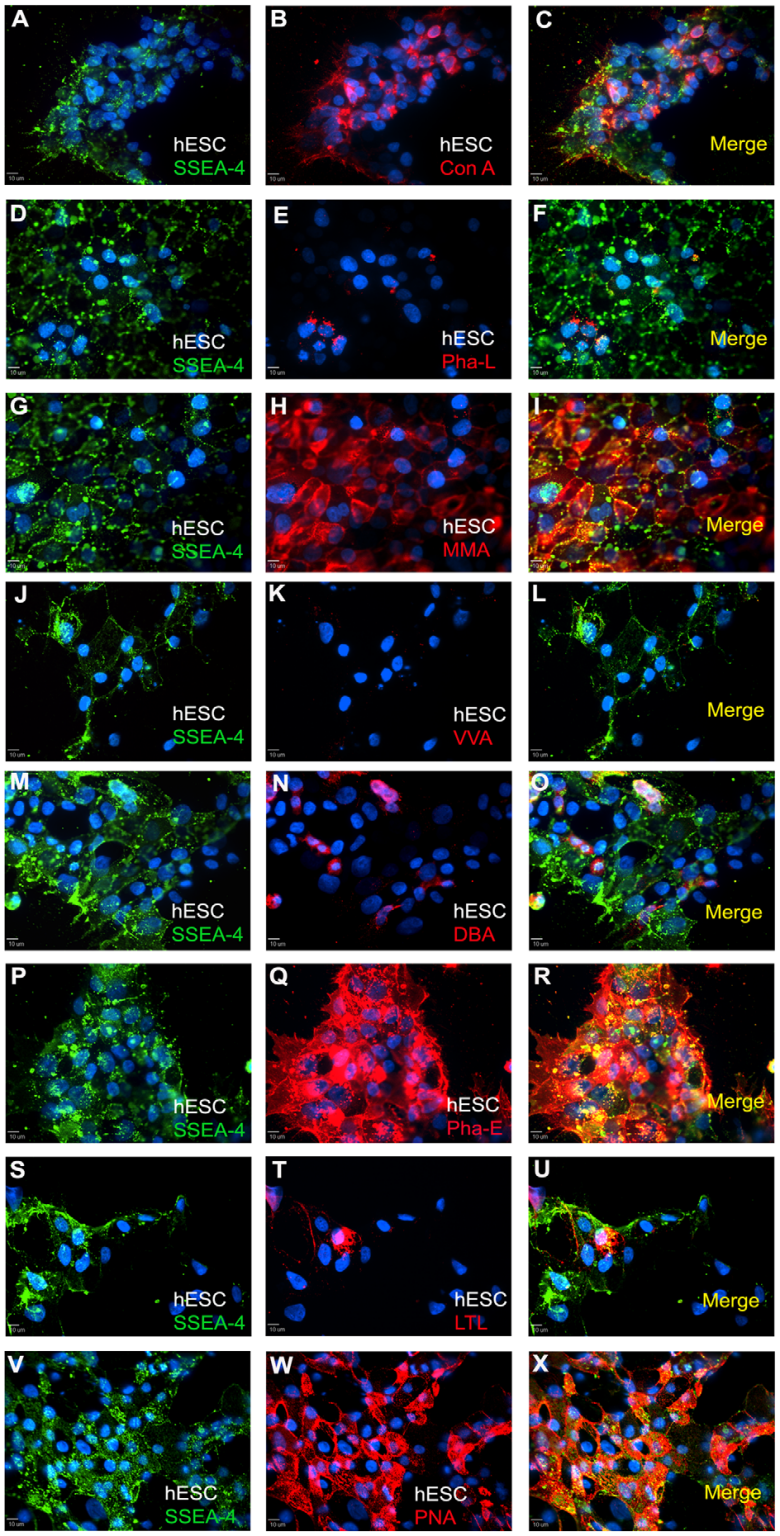
Immunocytochemistry of hESC cultures for SSEA-4 expression and binding to lectins. The panels in the left column show hESCs staining for SSEA-4 and DAPI (panels A, D, G, J, M, P, S, and V). In the same field of view as the left panel, the middle panels show binding of ConA(B), Pha-L (E), MMA (H), VVA (K), DBA (N), Pha-E (Q), LTL (T), and PNA (W) lectins in hESCs. The merge view of lectin and SSEA-4 staining is shown in panels in the right column. The staining amount roughly correlates with flow cytometry analysis. Scale bar: 10 µm.

Human ESCs, hNP cells and hMP cells were also analyzed by immunocytochemistry to determine localization of carbohydrates and to determine whether particular staining patterns were present in adherent cultures. Results for all 8 lectins supported our flow cytometry analysis. For hESCs, the cultures were co-stained with the pluripotency cell surface marker SSEA-4 (Figure 4, left column). ConA (Figure 4B), MAA (Figure 4H), PHA-E (Figure 4Q) and PNA (Figure 4W) appeared to bind uniformly to hES cell surface, and the intensity of staining was highest for PHA-E. The lectins PHA-L (Figure 4E), VVA (Figure 4K), DBA (Figure 4N) and LTL (Figure 4T) bound to only a small percentage of hESCs. The staining was punctuated but uniform throughout.

**Figure 5:**
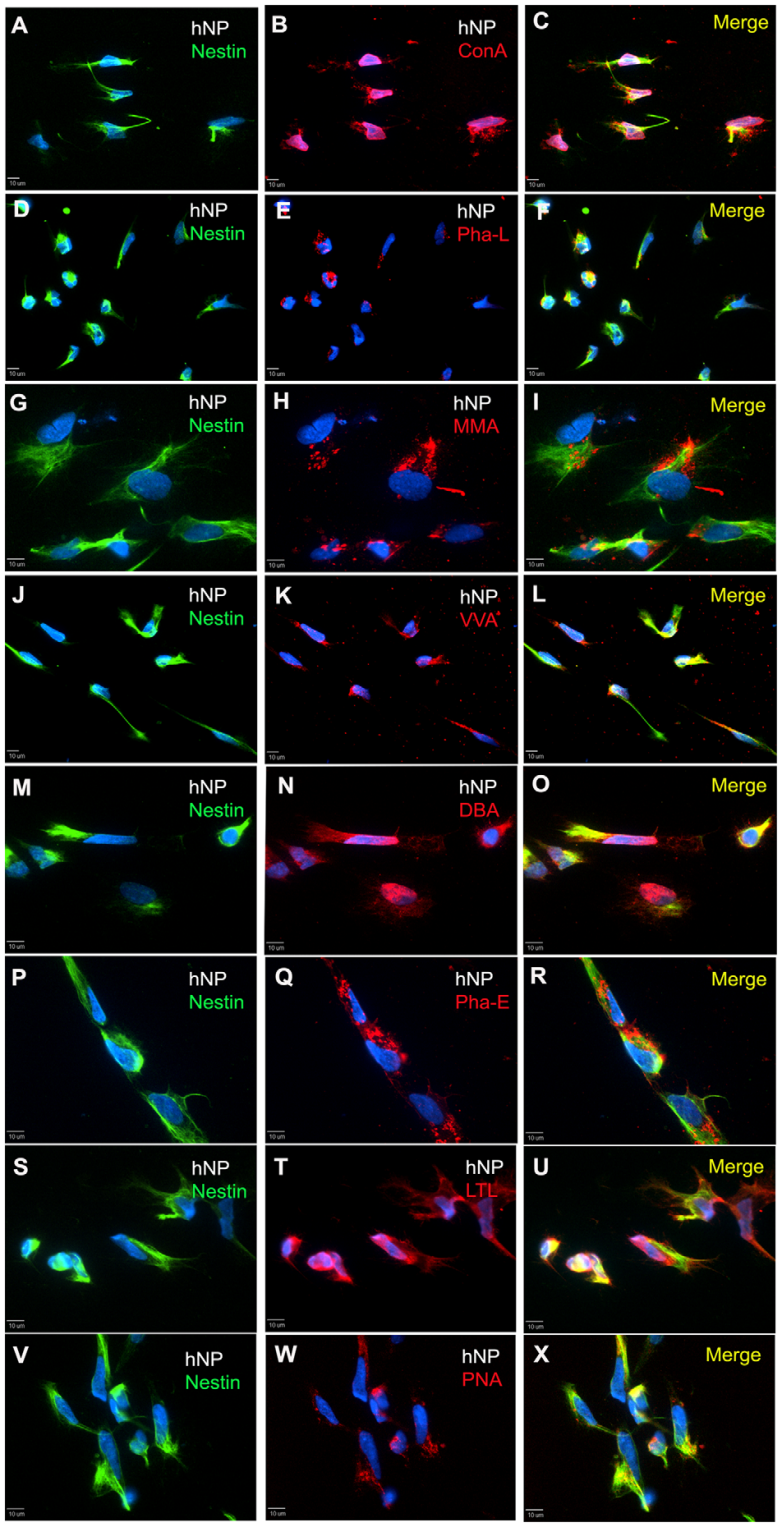
Immunocytochemistry of hNP cell cultures for Nestin expression and binding to lectins. The panels in the left column show hNP cells staining for Nestin and DAPI (panels A, D, G, J, M, P, S, and V). In the same field of view as the left panel, the middle panels show binding of ConA(B), Pha-L (E), MMA (H), VVA (K), DBA (N), Pha-E (Q), LTL (T), and PNA (W) lectins in hNP cells. The merge view of lectin and Nestin staining is shown in panels in the right column. The staining amount roughly correlates with flow cytometry analysis. Scale bar: 10 µm.

**Figure 6:**
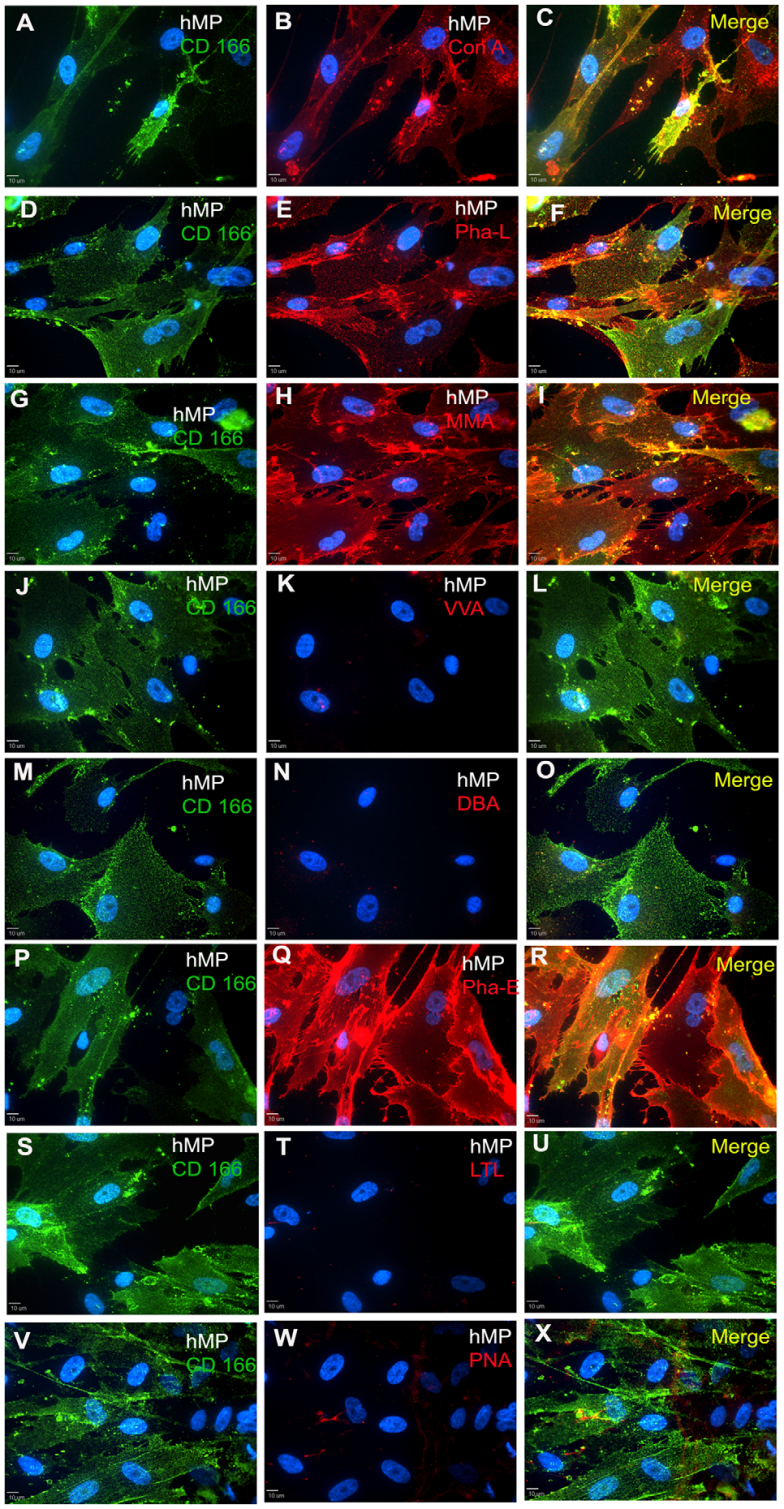
Immunocytochemistry of hMP cell cultures for CD 166 expression and binding to lectins. Binding of ConA(B), Pha-L (E), MMA (H), VVA(K), DBA(N), Pha-E (Q), LTL (T), and PNA (W) lectins in hMP cells. In the same field of view, CD 166 and DAPI staining is shown in panels in the left column. The merge view of lectin and CD 166 staining is shown in panels in the right column. The staining amount roughly correlates with flow cytometry analysis. Scale bar: 10 µm.

For hNP cells, all the lectins appear to bind uniformly to hNP cell surface, and the staining intensities appeared similar for all the lectins (Figure 5). hNP cells were co-stained for Nestin, a filament protein, and for the cell nuclei with DAPI, a DNA binding dye. We did not observe localization of lectin binding on regions on individual cells, cell clusters or cell phenotype. For hMP cells, high percentages of cells bound to ConA, PHA-L, MAA and PHA-E lectins (Figure 6B, 6E, 6H and 6Q, respectively), similar to our observations with flow cytometry. The lectin staining was punctuated and uniform all over the cell surface. Intensity of staining was low for ConA and highest for PHA-E lectin. The rest of the 4 lectins: VVA, DBA, LTL and PNA (Figure 6K, 6N, 6T and 6W, respectively) had very low binding with hMP cells, which corroborated with flow cytometry data. The hMP cells were co-stained for CD166, a mesenchymal marker, and cell nuclei with DAPI (Figure 6, left column).

### Fluorescence Assisted Cell Sorting of hNP cells using VVA-fluorescein lectin

**Figure 7:**
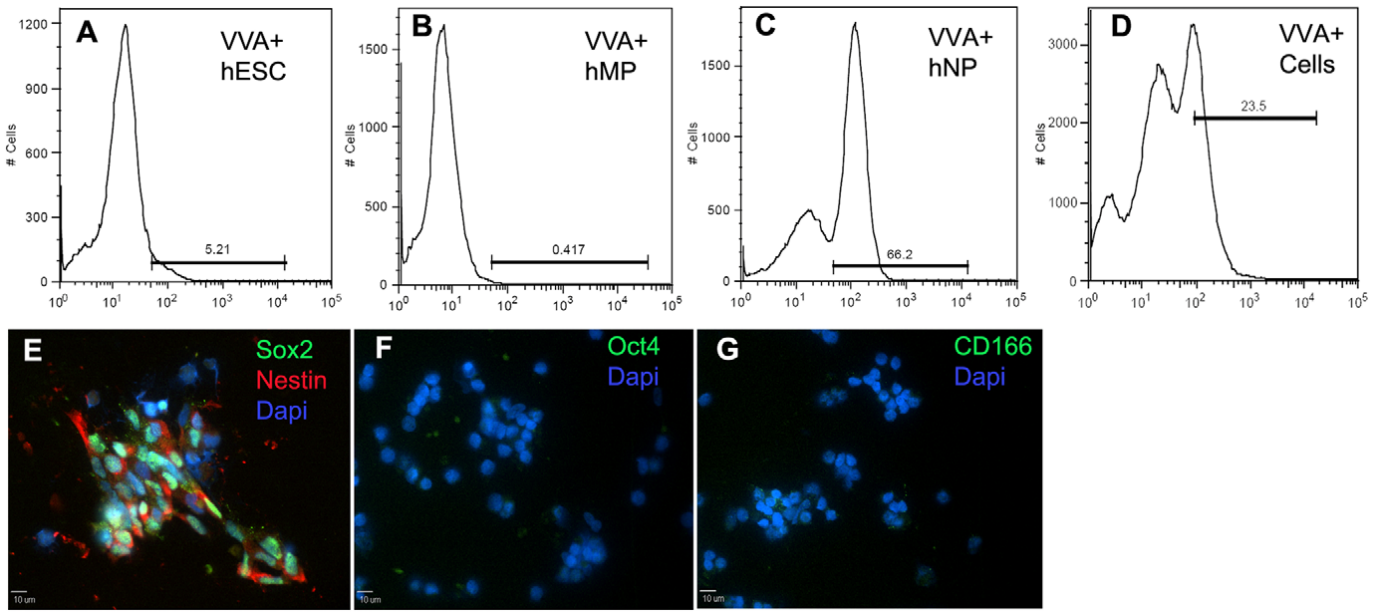
Fluorescence assisted cell sorting of hNPs using VVA lectin. hMP cells (A), hESCs (B) and hNP cells (C) stained with VVA-fluorescein lectin, analyzed for binding and used as controls. A mixed population of hESCs, hNPs and hMPs (1∶1∶1 ratio) was stained with VVA-fluorescein lectin and sorted for fluorescein positive cells (D). The sorted cells stained positive for Nestin and SOX2 (E); and were negative for OCT4 (F), and CD166 (G). Cells were stained with DAPI for nucleus. Scale bar: 10 µm.

VVA lectin bound to high percentages of hNP cells (96±3%), and low percentages of hESCs (1±0.3%) and hMP cells (1±0.1%) (Figure 3). Here we tested whether VVA lectin could be used to select hNP cells from a mix population of hESCs, hNP cells and hMP cells. The 3 cell types were harvested and mixed in 1∶1∶1 ratio; stained with VVA-fluorescein lectin and sorted for cells with highest fluorescein expression. The next day (approximately 16 hours later), the sorted cells were fixed and stained for expression of Nestin, SOX-2, Oct4, SSEA4 and CD 166 proteins. All the sorted cells (Figure 7) were positive for expression of Nestin, SOX-2, and negative for expression of Oct4 and CD166. These results indicate that VVA lectin binding can be used to isolate hNP cells from other cell types.

## Discussion

Since the isolation of hESCs, many studies have looked at the factors that cause or promote differentiation of hESCs. Most research is centered on the proteinous elements involved in differentiation while significantly less is being focused on the role of carbohydrate surface. Here, we probed the carbohydrate surface of WA09 hESCs and two of its derivative cell types, neural progenitors and mesenchymal progenitors, using lectins. A panel of 8 lectins was used which, although not exhaustive, allowed us to detect differences in glycan expressions among the 3 cell types. In this study we have identifed three glycan structures that are specifically upregulated in hNP cells as compared to hESCs and hMP cells. Further, based on one of the glycan structures, N-acetyl-D-galactosamine (binding to VVA lectin), we could isolate pure populations of hNP cells from a mix of hESCs, hNP cells and hMP cells. To our knowledge, this is the first study where glycan expression of hESCs, hNP cells and hMP cells has been compared to identify lineage specific expression. Also, this is the first study where VVA lectin has been used for isolation of hNP cells.

Transmembrane and cell surface proteins containing key carbohydrates are thought to play critical roles in neural stem cell biology including differentiation, migration, and cell renewal [21], suggesting the carbohydrate surface as one important extrinsic factor of neurogenesis. Importantly, recent research has been reported on various chondroitin sulfate proteoglycans (CSPGs), including aggrecan, phosphacan, and tenascin that are found in mouse neural precursor cells [22], and are similar to human CSPGs.

Previously, we have used lectins to catalog the various glycans presented on the carbohydrate surface of pluripotent, SSEA-4 sorted BG01 hESCs [16]–[17]. Wearne et al., using similar methodology, substantiated these previous results and discerned carbohydrate changes on BG01 hESCs allowed to randomly differentiate into 12- and 28- day embryoid bodies [18]–[19]. Various differentiated cell types were present in the embryoid bodies as determined by lineage specific markers. However, due to the minimal number of each differentiated cell type per lineage, glycosylation changes accompanying differentiation was limited. Here, we present the analysis of lectin binding and the glycan profiles of WA09 hESCs, hNP cells and hMP cells.

First, we characterized the hESCs, hNP cells and hMP cells with stem cell markers. Immunocytochemistry shows that WA09 hESCs are positive for embryonic stem cell markers Oct4, SSEA-4 and SOX-2, while negative for lineage specific markers Nestin (neural) and CD166 (mesenchymal). hNP cells were negative for Oct4, SSEA-4 and CD166, while positive for Nestin and SOX-2. Human NP cultures also lack expression of beta III tubulin (TUJ1), a marker for more mature neuronal cells. Our hNP cultures, when further differentiated into mature CNS cell types; do express markers for neurons, oligodendrocytes, and glial cells [1], [23]–[24]. Nestin, a widely referenced neuronal stem cell marker [25] was employed to stain in concert with each lectin. Nestin expression appears to be tightly regulated and after multipotent neural stem cells differentiate into a committed cell fate, Nestin expression decreases [26]. Similarly, hMP cells were negative for Oct4, SSEA-4 and SOX-2, while positive for Nestin and CD166. hMP cells used in this study can undergo osteogenic and chondrogenic, but not adipogenic differentiation; suggesting that hMP cells are multipotent progenitor cells [2].

A comparison of lectin binding of the multipotent hNP cells and hMP cells to the SSEA-4^+^ pluripotent hESCs enables the identification of glycan changes accompanying differentiation. PHA-L lectin binding, hence expression of N-type glycans containing β1–6 linked branches, was significantly up regulated upon hNP and hMP differentiation; suggesting that these glycan structures are important in differentiation. Potentially, PHA-L binding can be used to separate differentiated cell types from pluripotent hESCs and hence for enrichment of pluripotent cells. Using the 8 lectins mentioned in this study, we did not detect any glycan structures that were downregulated in both hNP cells and hMP cells, compared to hESCs. In future studies, we plan to use a more extensive collection of lectins to identify additional glycan structures that might be upregulated in hESCs and down regulated upon differentiation. Lectin microarray analysis can be used for high-throughput and sensitive analysis of a large set of biological samples and glycans [27]–[28]. Toyoda et al used lectin microarray technology to study glycan expression on undifferentiated hES cells and differentiated hES cells after embryoid body (EB) formation [28]. Similarly, glycan expression was compared between human induced pluripotent stem (hiPSC) cells, the parental lung fibroblasts they were generated from, and the differentiated hiPS EBs. The signals for MAL and PHA-L lectin binding in the hES cells were lower than those in EBs, while the signal for EEL is higher in hES cells than EBs. Hence EEL and PHA-L can be used for positive and negative identification, respectively, of hES cells. These findings corroborate with our results that PHA-L binding increases upon differentiation of hES cells. Hence, PHA-L can be used for exclusion of differentiated pluripotent stem cells. In a similar study Tateno et al compared glycan expression among 114 types of hiPSCs generated from five different somatic cells and compared their glycomes with those of hESC [27]. Lectin binding of PHA-E, PHA-L, VVAII lectins, among some others, decreases upon induction of pluripotency in somatic cells. This again is similar to our findings that binding of PHA-E, PHA-L and VVA is higher in hNP and hMP cells, compared to WA09 hESC.

Upon directed differentiation of hESCs into hNP cells, the lectins bind to higher percentage of hNP cells than hESCs (Figure 3). Conclusions from this work suggest the presence of many glycans on hNP surfaces. Expression of 3 glycans Neu5AC α(2,3) Gal/GalNAc (MAA), β GlcNac (PHA-E) and terminal β-Gal (PNA), is similar to hESCs. However, the expression of four glycans was significantly up regulated in hNP cells compared to hESCs: β1–6 branched oligosaccharides (PHA-L), GalNac end groups (VVA), α-linked N-acetylgalactosamine (DBA), and fucose moieties α-linked to GlcNAc (LTL). hNP cells' binding to VVA lectin is significantly higher than hESCs and hMP cells. Versican, a chondroitin sulphate proteoglycan and member of the lectican family, has been shown to be a major receptor for VVA lectin [29]. Versican induces neural differentiation and promotes neurite outgrowth [30]. Although we cannot be certain that VVA lectin is binding to versican, per se, we do see an increase in binding percentage of this lectin in neural differentiation, suggesting that GalNac end groups (VVA) might have utility as a glycan marker for neurogenesis. Further isolation, culture, and *in vitro* and *in vivo* differentiation of VVA^+^ hNP cells will determine the unique properties of this population of neural progenitor cells. Mandai et al demonstrated that combination of WGA and ECA lectins can be used to improve derivation of retinal photoreceptor cells from mouse ESC [31]. WGA and ECA lectins bind to Rx-positive cells and can be used for enrichment. The enrichment of Rx-positive cells significantly increased the further derivation of retinal photoreceptor precursor cells. It is possible that VVA lectins bind to a subtype of neural cells and could be used for their enrichment.

Whether the carbohydrate signature of cell type changes as a result of differentiation, or if glycans influence or play a more direct role in cellular fate is a question that plagues the field. Hence, it is critical to define the cell surface of differentiated cell types to probe glycan functions and potentially for cell sorting. In the present study we have identified glycan expression on homogenous populations of hESCs, hNP cells and hMP cells, and compared the glycan expression across the cell types. By comparing hESCs, hNP cells and hMP cells, we have identified glycan structures that are unique to hNP cells: GalNac end groups (VVA), α-linked N-acetylgalactosamine (DBA), and fucose moieties α-linked to GlcNAc (LTL). Future studies help in identifying the roles of these glycans in cell maintenance, proliferation and differentiation fate.
